# Uncinate fasciculus microstructural organisation and emotion recognition in schizophrenia: controlling for hit rate bias

**DOI:** 10.3389/fnbeh.2024.1302916

**Published:** 2024-03-19

**Authors:** Matthew Stevens, Síle Ní Mhurchú, Emma Corley, Ciara Egan, Brian Hallahan, Colm McDonald, Gary Donohoe, Tom Burke

**Affiliations:** ^1^School of Psychology, University of Galway, Galway, Ireland; ^2^Centre for Neuroimaging Cognition and Genomics (NICOG), University of Galway, Galway, Ireland; ^3^Clinical Neuroimaging Laboratory, Galway Neuroscience Centre, College of Medicine Nursing and Health Sciences, University of Galway, Galway, Ireland

**Keywords:** cognition, uncinate fasciculus, schizophrenia, emotion recognition, unbiased hit rate, childhood trauma

## Abstract

**Introduction:**

Schizophrenia (SCZ) is a complex neurodevelopmental disorder characterised by functional and structural brain dysconnectivity and disturbances in perception, cognition, emotion, and social functioning. In the present study, we investigated whether the microstructural organisation of the uncinate fasciculus (UF) was associated with emotion recognition (ER) performance. Additionally, we investigated the usefulness of an unbiased hit rate (UHR) score to control for response biases (i.e., participant guessing) during an emotion recognition task (ERT).

**Methods:**

Fifty-eight individuals diagnosed with SCZ were included. The CANTAB ERT was used to measure social cognition. Specific ROI manual tract segmentation was completed using ExploreDTI and followed the protocol previously outlined by Coad et al. (2020).

**Results:**

We found that the microstructural organisation of the UF was significantly correlated with physical neglect and ER outcomes. Furthermore, we found that the UHR score was more sensitive to ERT subscale emotion items than the standard HR score. Finally, given the association between childhood trauma (in particular childhood neglect) and social cognition in SCZ, a mediation analysis found evidence that microstructural alterations of the UF mediated an association between childhood trauma and social cognitive performance.

**Discussion:**

The mediating role of microstructural alterations in the UF on the association between childhood trauma and social cognitive performance suggests that early life adversity impacts both brain development and social cognitive outcomes for people with SCZ. Limitations of the present study include the restricted ability of the tensor model to correctly assess multi-directionality at regions where fibre populations intersect.

## Introduction

### Schizophrenia

Schizophrenia (SCZ) is a complex and debilitating neurodevelopmental disorder characterised by positive (i.e., hallucinations and delusions), negative (i.e., flat affect and avolition), and cognitive symptoms (i.e., memory impairment and social cognition deficits) (Mueser and McGurk, 2004; Tripathi et al., 2018; Correll and Schooler, 2020; McCutcheon et al., 2023). SCZ poses a significant risk to a person’s functional, occupational, social, psychological, and life outcomes, i.e., mortality rates (Lepage et al., 2014; Davis et al., 2016; Correll et al., 2022). Meta-analytic evidence suggests that across the lifespan, positive symptoms tend to vary over time, negative symptoms generally persist, cognitive impairments remain consistent and often predate illness onset, and declines in both grey and white matter (WM) volume are evident relative to controls (Lepage et al., 2014; Heilbronner et al., 2016).

Key areas of interest in the aetiology of SCZ are the influence of genetics, including the dopamine receptor genes DRD2 and DISC1, environment, i.e., psychosocial stressors, gene–environment interactions, and the timing of these interactions (Gejman et al., 2011; He et al., 2016; Dahoun et al., 2017; Torrey and Yolken, 2019; Wahbeh and Avramopoulos, 2021). Hilker et al. (2018) found concordance rates of up to 40% for monozygotic (MZ) twins, leaving the remaining 60% potentially attributable to environmental components (Prescott and Kendler, 1995; Kringlen, 2000) such as cannabis use, childhood trauma, ethnic density, and related psychosocial stressors such as social inequality (Krabbendam and van Os, 2005; Veling et al., 2008; Bourque et al., 2011; Shaw et al., 2012; Popovic et al., 2019; Correll and Schooler, 2020). As for the impact of childhood trauma, it has been associated with poorer social cognitive outcomes such as impaired emotion recognition (ER) in several psychiatric conditions such as SCZ (Garcia et al., 2016; Rokita et al., 2018). However, the Childhood Trauma Questionnaire (CTQ) facet of physical neglect has been most strongly associated with impairments in cognition in patients with SCZ (Vaskinn et al., 2020; King et al., 2021; Rokita et al., 2021). The complex interaction of the above key areas has been considered through the diathesis-stress model, which proposes that certain individuals are at a higher risk of developing a mental health condition due to genetic predisposition combined with exposure to environmental stressors (Walker and Diforio, 1997). Whereas the timing of these complex interactions has been considered through a ‘multiple-hit’ theory that has been variously articulated, exposure to environmental stressors during critical neurodevelopmental windows may disrupt and alter later brain maturation processes in those genetically susceptible (Davis et al., 2016; van der Meer et al., 2022).

### White matter abnormalities in schizophrenia

Alterations in WM have been robustly reported in individuals with SCZ (Kubicki et al., 2005), with extensive decreases found in projection, commissural, and association WM tracts (Kelly et al., 2018). The uncinate fasciculus (UF) is a long-range WM association tract of the brain, connecting the medial orbitofrontal cortex with the anterior temporal lobes (Gilchrist and Thompson, 2021). The UF is essential for several processes such as episodic memory, language, visual associative learning, and social and emotional functioning (Von Der Heide et al., 2013; Olson et al., 2015; Ben-Soussan et al., 2020; Catani, 2022; Semenza, 2022). In a large-scale meta-analytic study of four separate psychiatric conditions, reductions in fractional anisotropy (FA) of the UF tract were found to be specific to people with SCZ (Koshiyama et al., 2020). The UF has also been linked to mentalising and ER, a deficit that is found in people with SCZ (Coad et al., 2020; Zhao et al., 2021). UF tract abnormalities have also been directly associated with social and emotional impairments in children with autism spectrum disorder (ASD) (Li et al., 2019).

### Social cognition in schizophrenia

Social cognition is a broad concept that refers to the ability to assess and recognise facial expressions and emotions, to observe others for social referencing and cues, empathy, and theory of mind, e.g., the ability to understand what others may be thinking and/or feeling and social context (Leslie et al., 2004; Frith, 2008). Research has demonstrated that worse performance during an emotion recognition task (ERT), specifically for neutral expressions, predicted the transition to psychosis in people at ultra-high-risk for psychosis (Allott et al., 2014; Davis et al., 2016).

Emotion recognition task scores provide a ‘total hit rate’ (HR) representing the total amount of correct responses, i.e., the participant selects ‘angry’ when an angry face is presented; however, the use of this total score may misrepresent accurate scores for participants who may be guessing their responses, i.e., the participant selects angry for every trial, therefore achieving 100% accuracy for the anger subset (Wagner, 1993). The unbiased hit rate (UHR) accounts for some response biases (i.e., single-item strategizing or guessing) that consider the number of correct answers for the target emotion, the number of times the target emotion appeared, and the total number of times the participant selects the target emotion as an answer (both correct and incorrect answers) (Wagner, 1993; Griffiths et al., 2015). As individuals with SCZ present with ER deficits, and it has been shown to be associated with clinical symptoms, the UHR may be more fruitful than a regular HR for assessing the true extent of an ER deficit (Wagner, 1993; Kohler et al., 2010; Davis et al., 2016).

### Uncinate fasciculus and emotion recognition

Previous research has demonstrated links between FA of the UF and social cognitive domains such as ER in typical control or ‘healthy’ participants (Anderson et al., 2015; Unger et al., 2016; Coad et al., 2020). Coad et al. (2020) investigated ER correlates with UF FA in 86 self-reported healthy participants and reported significant moderately positive correlations between FA of the right UF and emotional (*p* = 0.003, *r* = 0.416), but not neutral facial expression (*p* = 0.074, *r* = 0.227) recognition. Furthermore, FA of the right UF showed significant moderate positive correlations with positive valanced items (*p* = 0.005, *r* = 0.422) but not negative valanced items (*p* = 0.093, *r* = 0.263) (Coad et al., 2020). In a study involving 49 healthy preschool children, Anderson et al. (2015) reported significant positive correlations between FA of the left UF and performance on a mental state inference test in 4-year-old’s (*p* < 0.01, *r* = 0.63) but not in 6-year-old’s (*p* = 0.68, *r* = 0.08). Conversely, one study by Unger et al. (2016) involving 28 neurologically healthy participants found non-significant relationships between UF FA and individual variations in ER performance. Moxon-Emre et al. (2019) investigated ER performance in 36 children treated for posterior fossa (PF) tumours and 16 healthy controls. They found that FA in the left UF correlated with and predicted greater ER performance, but only in healthy participants (*p* < 0.05, *r* > 0.63). In adults following glioblastoma surgery, it has been reported that lower FA in the right UF was significantly and positively correlated with lower ER performance post-surgery (Sinha et al., 2020). In a study of 33 individuals with moderate–severe traumatic brain injury (TBI), significant moderate positive correlations (*r* = 0.6, *p* < 0.001) were also found between FA of the left UF and ER (Rigon et al., 2019). Within dementia syndromes, Multani et al. (2017) supported these findings in individuals with primary progressive aphasia (PPA), reporting significant moderate positive correlations (*r* = 0.50, *p* = 0.001) between FA of the right UF and ER performance. A further study involving 16 patients with amnestic mild cognitive impairment (aMCI) found significant moderate positive correlations between FA of the left UF and ER subscales of sadness (*r =* 0.536, *p* = 0.033) and surprise (*r* = 0.509, *p* < 0.044), which were non-significant following multi-comparison control (Fujie et al., 2008). Crespi et al. (2016) conducted a study including individuals with amyotrophic lateral sclerosis (ALS) and reported significant partial positive correlations (*r* = 0.79, *p* < 0.0001) between the mean FA of the right UF and performance on an emotional attribution story-based task.

In contrast to this collective of findings across healthy controls, neurosurgical patients, and people with neurodegenerative conditions, Jung et al. (2020) found that reduced FA of the right UF had significant moderate negative correlations (*r* = −0.372, *p* = 0.017) with the social–emotional perception of self and other in individuals with psychosis.

Both left and right UF relate to ER outcomes. However, some conflicting directional differences in correlation analysis findings of FA in the UF and ER (i.e., Jung et al., 2020; Sinha et al., 2020) suggest that further research is required to investigate bilateral UF FA associations with ER and the direction of these associations if these associations are lateralized to the left or right hemisphere. Moreover, few studies have investigated the relationship between bilateral UF FA and ER in individuals with SCZ, and to our present knowledge, no studies have investigated the utility of a UHR score in detecting ER deficits in individuals with SCZ.

### The current study

The present study aimed to investigate the relationship between the UF and precise ER measurement outcomes. As above, physical neglect is the facet of the CTQ most associated with cognitive impairment in SCZ (Rokita et al., 2018, 2021; King et al., 2021), and so we further hypothesised that FA of the UF would both correlate with and predict ER outcomes and postulated that FA of the UF would mediate the relationship between childhood trauma, specifically physical neglect, and ER outcomes. The focus of ER ability is of interest as it is important for understanding others’ emotions and intentions, and deficits in this ability have been extensively linked to SCZ and psychotic symptoms (Gao et al., 2021). These impairments in individuals with SCZ may cause significant interpersonal distress and influence delusion formation via the misunderstanding of others’ intentions and the misattribution of emotions to neutral facial expressions in social settings, thereby contributing to the onset of psychotic symptoms (Blackwood et al., 2001; Davis et al., 2016; Gao et al., 2021). Allott et al. (2014) demonstrated that impaired ability to recognise neutral facial expressions predicted transition to psychosis in individuals at ultra-high risk for psychosis. ER impairments have also been associated with disorganised thinking, poorer social and emotional functioning, and partially aggressive behaviour in individuals with SCZ (Addington et al., 2006; Yildirim et al., 2018; Bulgari et al., 2020).

Childhood physical neglect, which ultimately signals childhood deprivation, is also of interest as it has been specifically linked to poorer cognitive performance (Üçok et al., 2015; Li et al., 2017; Mørkved et al., 2020; Vaskinn et al., 2021; Lakkireddy et al., 2022), as well as altered WM connectivity, including FA of the UF (Govindan et al., 2010; Hanson et al., 2015; Tendolkar et al., 2018). The UF is of interest as previous studies have shown specific links between the microstructural organisation of the UF and ER performance (Coad et al., 2020). Furthermore, the UF has specifically demonstrated stronger associations with ER performance (particularly in the right hemisphere) when compared to a control tract, the CST (Coad et al., 2020). Early childhood neglect has also been shown to impact the CST, resulting in lower FA in adolescents who experienced early childhood neglect (Hanson et al., 2015). Importantly, childhood physical neglect may also hinder a person’s ability to accurately recognise basic facial emotional expressions and increase their likelihood of misattributing negative emotions to neutral facial expressions (Pollak et al., 2000). As mentioned previously, several studies have linked the FA of the UF to ER performance outcomes in various populations, with few including individuals with SCZ (Fujie et al., 2008; Coad et al., 2020; Jung et al., 2020). Of the studies that included individuals with SCZ, to our knowledge, none have assessed the utility of a UHR for detecting ER impairments in individuals with SCZ. Thus, the present study aimed to further investigate the link between the microstructural organisation of the UF and ER performance and whether physical neglect in childhood mediated this relationship in individuals with SCZ whilst controlling for hit rate (HR) bias. This study was preregistered prior to UF tract extraction and data analysis (AsPredicted#137730).

## Methods

### Ethical approval

Ethical approval was granted from the University of Galway, Galway University Hospitals and Tallaght Hospital Dublin Research Ethics Committees. All participants provided written informed consent.

### Participants

Of the total sample recruited to *iRELATE*, *n* = 58 patients with SCZ, aged between 18 and 63 years, completed neuroimaging. All participants had a previously established clinical diagnosis of SCZ or schizoaffective disorder as outlined by the Diagnostic and Statistical Manual of Mental Disorders (DSM-IV; American Psychiatric Association, 2000). At assessment, the diagnosis was confirmed by the Structured Clinical Interview for DSM-IV (SCID, First and Gibbon, 2004). Patients were recruited from outpatient hospital departments and mental health services in Ireland. Exclusion criteria included: history of an acquired brain injury; historical or comorbid neurological or Axis I psychiatric condition; previous significant loss of consciousness requiring medical attention; intellectual disability; or a history of substance misuse disorder 6 months prior to study commencement. Contraindications for undergoing MRI scanning, i.e., the presence of a metal implant or device, were also exclusion criteria.

### MRI acquisition

Diffusion magnetic resonance imaging (MRI) was collected for this project. An initial MRI scan was completed on all participants before disseminating diffusion data. The standard structural MRI scan was completed using a 3 T Philips Achieva MR scanner. The scanner is located in the Centre for Advanced Medical Imaging at St. James’ Hospital in Dublin 8, Ireland. Each participant underwent a structural whole-brain MRI scan which followed a pre-determined acquisition sequence including three-dimensional T1-weighted images using a ‘Fast Field Echo’ pulse sequence with a spatial resolution of 1 mm^3^. The scan ‘Repetition Time’ (TR) was 8.5 ms, and ‘Echo Time’ (TE) was 3.9 ms. The ‘Inversion Time’ from the time elapsed between pulses was 1,060 ms with a ‘Flip Angle’ of 8^0^. The acquisition sequence was obtained in millimetres over a distance (field of view) of 256 × 256 × 160 mm^3^, and the acquisition time was 7 min and 30 s in total. Foam padding was used to preserve a secured head position for the duration of the MRI scan, and participants were supplied with headphones to dampen noise interference.

### DTI extraction

Pre-processing steps involve corrections and quality control (QC) steps, including eddy current echo-planar imaging corrections, which can both result in image distortion during diffusion imaging (Bodammer et al., 2004). Tensor fitting was also included as a QC step. The primary outcome of FA was established, and the UF and cortico-spinal tract (CST) were extracted as the specific region of interest (ROI). The CST was considered in this study to act as a divergent validity marker, in line with previous research on UF and ER (Coad et al., 2020). Specific ROI manual tract segmentation was completed using ExploreDTI, in line with the protocol previously described by Coad et al. (2020) and outlined below.

### Uncinate fasciculus extraction protocol

**Figure 1:**
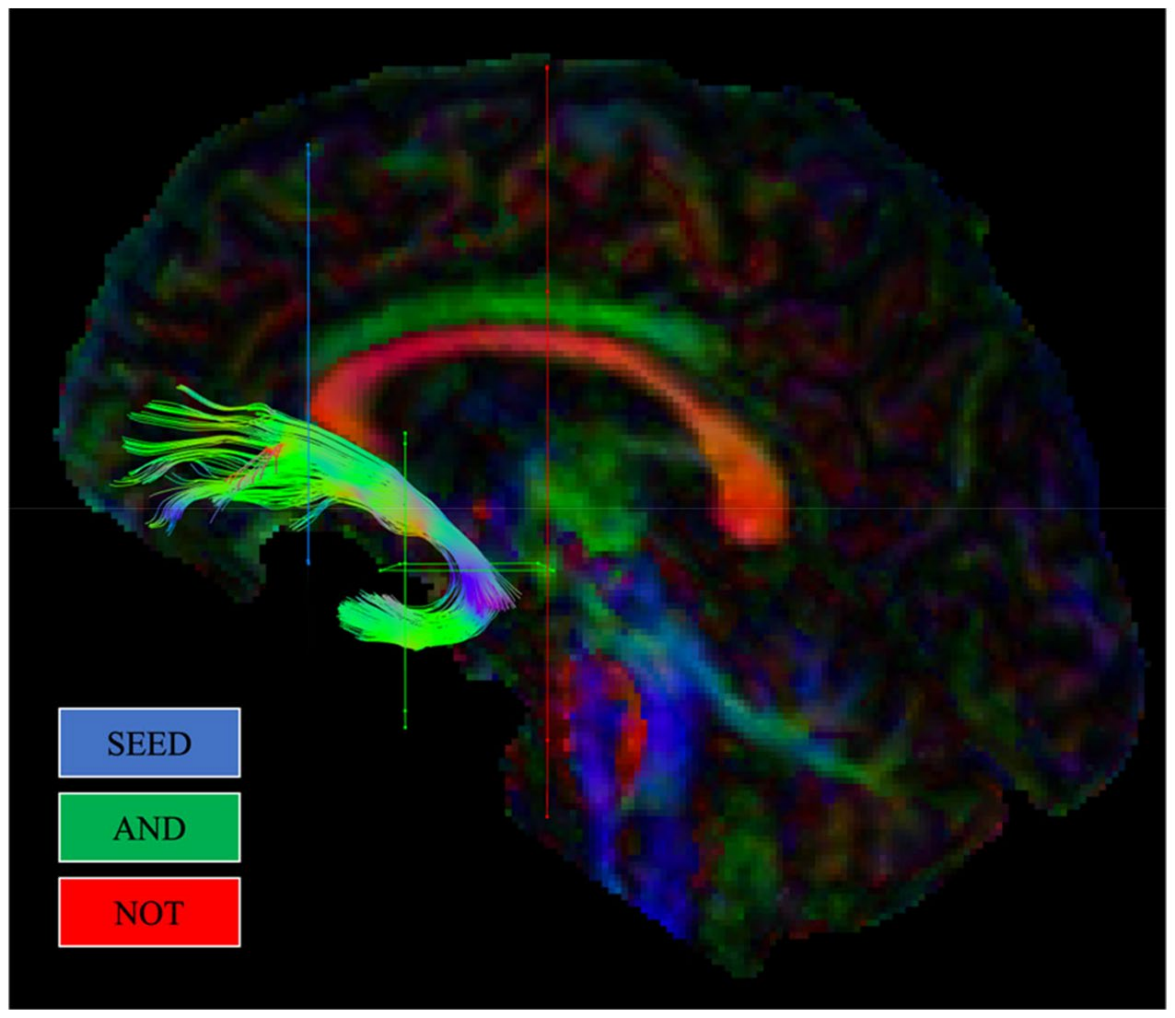
Example reconstruction of the uncinate fasciculus (UF) from a single participant, in line with Coad et al. (2020). Regions of interest gates (ROIs) used for reconstructing each tract are shown.

The protocol previously outlined by Coad et al. (2020) mapped the position of the UF based on a description from Catani and Thiebautdeschotten (2008). First, a SEED ROI was created on a coronal slice in the inferior medial area at the entrance of the UF into the frontal lobe, anterior to the corpus callosum. Second, two AND gates were created in the temporal lobes. The first AND gate was created on a coronal section situated anteriorly at the point where the temporal and frontal lobes connect. The second AND gate was created on an axial section aligned with the top section of the pons, encapsulating the location where the UF rotates around the Sylvian fissure. Two NOT gates were included. The first was created on a coronal section posterior to the pons. This gate encompassed the entire brain in order to remove unrelated fibres, such as the inferior fronto-occipital fasciculus (IFOF). The second NOT gate was created on a sagittal section of the brain, situated at the interhemispheric fissure. This NOT gate also spanned the entire brain to exclude irrelevant fibres from inclusion, such as the anterior commissure. Upon creation of the SEED, AND, and NOT gates, both left and right UF tracts were visually examined to assess the consistency of each tract with the established path of the UF. Furthermore, NOT gates were created to exclude irrelevant fibres that, upon visual examination, were discovered to be inconsistent with the established path of the UF. Figure 1 illustrates gate placement for the UF, in line with this protocol, using ‘SEED’ regions alongside ‘AND’ and ‘NOT’ gates.

### Corticospinal tract extraction protocol

The protocol previously outlined by Coad et al. (2020) mapped the position of the CST based on a description from Wakana et al. (2007). Two AND gates were created: the first was created on an axial section situated above the superior colliculus and spanned the whole cerebral peduncle on the left or right hemisphere—in line with the tract being extracted. The second AND gate was created on an axial section spanning the location where the CST separates to travel on each side of the central sulcus. Following on from the UF protocol, NOT gates were created to exclude irrelevant fibres that were discovered and inconsistent with the established path of the CST.

Fractional anisotropy values are used as a measure to index water diffusion directionality in WM fibre tracts and were used in the present study to assess WM microstructural organization. FA is sensitive to changes in biophysical tissue properties, such as alterations in axonal diameter, fibre density, and myelin structure (Basser and Pierpaoli, 2011). FA values are scaled from 0 to 1, whereby zero would reflect free-flowing unrestricted liquid, values greater than 0.2 signify tissue types such as WM, and values closer to 1 are associated with high levels of the microstructural organisation, such as dense major WM tracts in the brain.

### Emotion recognition task, cambridge neuropsychological test automated battery

The ERT from the CANTAB is a computerised task used to measure the ability to identify six basic emotions, namely sadness, happiness, anger, disgust, surprise, and fear (Robbins et al., 1998). Participants are asked to choose one out of six emotions describing the emotional expression presented on the screen. In many studies, total accuracy or ‘Total Hits’ is the primary outcome. This is a simple tally of correctly identified stimuli. The UHR is calculated for each participant, per emotional subtype, i.e., happy, sad, and angry, and incorporates the correct responses for a target emotion by a participant (i.e., the hits or correct response), the number of stimuli representing this emotion (how many times it was displayed), and the overall frequency of this emotion category being chosen (the number of times it was chosen, regardless of accuracy). The UHR has a scaled range from 0 to 1, with a score closer to 1 indicating whether all stimuli for a target emotion have been correctly identified in line with the above formula.

### Positive and negative syndrome scale

The Positive and Negative Syndrome Scale (PANSS; Kay et al., 1987) was used to assess the symptoms and severity of SCZ. The PANSS is a 30-item rating scale consisting of three scales: positive (7 items), negative (7 items), and general psychopathology (16 items). To reflect the ‘absence’ scores, we re-scaled the Likert scale to 0–6 so that 0 represented ‘absent ‘and 6 ‘high’. The total scores ranged from 0 to 42 for the positive and negative scales, 0 to 96 for the general scale, and 0 to 138 for the total score. Cronbach’s alpha for the PANSS has a range of 0.70–0.85 (Van den Oord et al., 2006).

### Childhood trauma questionnaire

The experience of childhood physical neglect was measured using the physical neglect subscale of the CTQ (Bernstein et al., 1994). The CTQ is a clinically valid tool that participants complete retrospectively. Each subscale includes five items, and individuals are asked to respond whether they had experienced the event on a Likert scale ranging from ‘1’ (‘never true’) to ‘5’ (‘very often true’). The CTQ has strong psychometric properties, as demonstrated in both clinical and non-clinical samples (Scher et al., 2001).

### Statistical analysis

The present study used a within-subjects design. Analyses were conducted utilising the Statistical Package for Social Sciences (SPSS) Version 28 (SPSS Inc., IBM, New York, United States), i.e., ANOVA, correlation, and linear regression analysis. Hemispheric structural differences were assessed using an ANOVA. Pearson’s correlation analysis was used to determine correlations between clinical, cognitive, demographic, and structural data. The outcome of this analysis matrix was used to determine the sensitivity and usefulness of a standard HR score or a UHR score. Linear regression analysis was then used to assess whether the UF structure directly predicted ER outcomes.

Finally, we carried out a left and right lateralised mediation analysis with physical neglect as a predictor variable, FA of the respective UF as a mediator, and ER as an outcome variable. The purpose was to determine whether childhood trauma was associated with UF microstructural organisation and whether that, in turn, was associated with social cognitive outcomes. For the purposes of comparative divergent validity, the CST was considered in line with the protocol of Coad et al. (2020). For the mediation analyses, a bootstrapping approach was implemented using the SPSS macro PROCESS V 4.2 (Hayes, 2012). This allows for the estimation of direct and indirect effects by applying an ordinary least squares path analytic framework. The significance of indirect effects is assumed if the 95% confidence interval (95% CI) does not include zero. The number of bootstrap samples was set to *n* = 5,000. Standardised beta regression coefficients and standard errors (SEs) are presented for each effect.

## Results

### Demographic and clinical information

The mean age of the participants was 42.94 ± 11.13, and 63.8% were men. Participants had a mean PANSS total score of 38.59 ± 2.23, a PANSS general score of 20.53 ± 4.08, a positive score of 8.59 ± 2.23, and a negative score of 9.71 ± 3.82. Participants had a mean CTQ subscale physical neglect score of 7.69 ± 3.31.

### Demographic and clinical correlates

A non-significant weak negative correlation between FA of the right UF and age, *r* = −0.280, and left UF, *r* = −0.200, was observed. The PANSS total score and FA of the left UF, *r* = 0.14, *p* = 0.341, and FA of the right UF, *r* = 0.01, *p* = 0.930 were not significantly related. Furthermore, no statistically significant relationships were found between FA of either UF and illness onset, illness duration, positive or negative symptoms, and/or the general PANSS subscale. Significant moderate negative correlations were also found between childhood physical neglect and FA of the left UF [*r*(50) = −0.379, *p* = 0.006] and right UF [*r*(50) = −0.437, *p* = 0.001].

### Structural comparisons

Comparing the whole structure, there was no significant difference between the FA of the left UF and the right, and they were strongly correlated (*r* = 0.686, *p* ≤ 0.001). Notwithstanding, when bilaterally segmented into anterior and posterior UF, there was a significant difference when FA of the anterior regions, but not posterior regions, were compared (*p* = 0.005).

### Emotion recognition task correlates

A Pearson’s correlation analysis was conducted to determine relationships between the left and right UF, ERT standard HR total scores, and subscale emotion items (see Table 1). The results indicated a moderately positive correlation between the standard HR total score and the left UF [*r*(50) = 0.448, *p* = <0.001] and the right UF [*r*(50) = 0.451, *p* = <0.001]. The results for subscale emotion items indicated moderately positive correlations between the left UF and anger [*r*(50) = 0.358, *p* = 0.009], and disgust [*r*(50) = 0.312, *p* = 0.024]. Moderate positive correlations were also found between the right UF and fear [*r*(50) = 0.328, *p* = 0.017], surprise [*r*(50) = 0.395, *p* = 0.004], and disgust [*r*(50) = 0.318, *p* = 0.022].

**Table 1:** Correlation coefficients for unbiased and total hits and subscale items.

Variable		UF Left	UF Right	Total ERT	Happiness	Sadness	Fear	Anger	Surprise	Disgust
UF Left	*r*	-	0.686^***^	0.428^**^	0.182	0.262	0.260	0.443^***^	0.333^*^	0.318^*^
	*p* value		<0.001	0.002	0.198	0.061	0.068	<0.001	0.016	0.022
UF Right	*r*	0.686^***^	-	0.420^**^	0.146	0.185	0.312^*^	0.368	0.494^***^	0.300^*^
	*p* value	<0.001		0.002	0.302	0.189	0.028	0.009	<0.001	0.031
Total ERT	*r*	0.448^***^	0.451^***^	-	0.629^***^	0.759^***^	0.589^***^	0.580^***^	0.701^***^	0.706^***^
	*p* value	<0.001	<0.001		<0.001	<0.001	<0.001	<0.001	<0.001	<0.001
Happiness	*r*	0.250	0.160	0.481^***^	-	0.468^***^	0.266	0.103	0.296^*^	0.206
	*p* value	0.074	0.256	<0.001		<0.001	0.062	0.477	0.033	0.143
Sadness	*r*	0.238	0.191	0.778^***^	0.388^**^	-	0.282^*^	0.409^**^	0.324^*^	0.456^***^
	*p* value	0.089	0.175	<0.001	0.004		0.047	0.003	0.019	<0.001
Fear	*r*	0.249	0.328^*^	0.559^***^	0.297^*^	0.335^*^	-	0.064	0.571^***^	0.196
	*p* value	0.076	0.017	<0.001	0.032	0.015		0.662	<0.001	0.173
Anger	*r*	0.358^**^	0.274	0.570^***^	0.152	0.327^*^	0.060	-	0.240	0.483^***^
	*p* value	0.009	0.050	<0.001	0.281	0.018	0.671		0.094	<0.001
Surprise	*r*	0.246	0.395^**^	0.507^***^	−0.087	0.207	0.189	0.217	-	0.431^***^
	*p* value	0.079	0.004	<0.001	0.538	0.140	0.181	0.123		<0.001
Disgust	*r*	0.312^*^	0.318^*^	0.721^***^	0.090	0.485^***^	0.212	0.360^**^	0.333^*^	-
	*p* value	0.024	0.022	<0.001	0.526	<0.001	0.131	0.009	0.016	

Upper portion relates to the ERT unbiased hit rate; lower portion relates to the ERT total hits; UF, Uncinate fasciculus; ERT, Emotion recognition task; ^*^p < 0.05; ^**^p < 0.01; ^***^p < 0.001; Bonferroni adjusted *p* < 0.005.

A Pearson’s correlation analysis was conducted to determine relationships between the left and right UF, ERT UHR total scores, and subscale emotion items (see Table 1). The results indicated a moderately positive correlation between the UHR total score and the left UF [*r*(50) = 0.428, *p* = 0.002] and the right UF [*r*(50) = 0.420, *p* = 0.002]. The results for subscale emotion items indicated moderate positive correlations between the left UF and anger [*r*(50) = 0.443, *p* = 0.001], surprise [*r*(50) = 0.333, *p* = 0.016], and disgust [*r*(50) = 0.318, *p* = 0.022]. Moderate positive correlations were also found between the right UF and anger [*r*(50) = 0.368, *p* = 0.009], surprise [*r*(50) = 0.494, *p* = <0.001], and disgust [*r*(50) = 0.300, *p* = 0.031], and fear [*r*(50) = 0.312, *p* = 0.028].

There were no significant relationships between the HR and UHR total scores and the PANSS total (*p* > 0.05). Additionally, there were no relationships between PANSS subscale items and HR/UHR subscale items (*p* > 0.05).

### Regression

Considering the UHR was observed to relate more than the HR to the microstructural organisation of the UF, linear regressions were conducted to determine whether FA of the UF significantly predicted the UHR total scores. FA of the left UF significantly predicted the UHR total score (β = 1.01, *t* = 3.32, *p* = 0.002), accounting for 18% of the variance [*R*^2^ = 0.18, *F*(1, 49) = 10.99, *p* = 0.002]. FA of the right UF significantly predicted UHR total score (β = 1.12, *t* = 3.24, *p* = 0.002), further accounting for 18% of the variance [*R*^2^ = 0.18, *F*(1, 49) = 10.52, *p* = 0.002]. For divergent validity, the CST was also considered, as above. There was no significant relationship between the UHR and CST.

### Mediation analysis

Mediation analyses were conducted to determine the independent role of the left and right UF microstructural organisations in the relationship between childhood physical neglect and UHR. Model 1 investigated the mediating role of the left UF, whilst Model 2 investigated the mediating role of the right UF. There was no significant association found for the CST.

**Figure 2:**
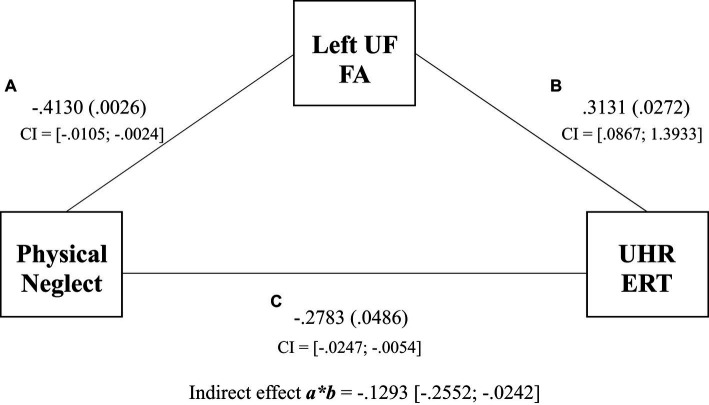
Mediation of physical neglect, left uncinate fasciculus, and the unbiased hit rate total regression coefficients and *p* values for associations between physical neglect, FA of the left UF, and UHR total scores. Indirect effect and confidence intervals are represented by a*b.

The results for Model 1 demonstrated a significant indirect effect of physical neglect on UHR ERT via FA in the left UF (β_indirect_ = −0.1293, SE = 0.0599, 95% CI [−0.2552 to −0.0242]) (see Figure 2). Physical neglect was significantly associated with FA in the left UF (β_direct_ = −0.4130, SE = 0.020, *p* = 0.0026), and the left UF was significantly associated with UHR ERT (β_direct_ = 0.3131, SE = 0.3249, *p* = 0.0272). Physical neglect was significantly associated with UHR ERT (β_direct_ = −0.2783, SE = 0.0051, *p* = 0.0486). The total effect of physical neglect on the UHR ERT was also significant (β = −0.0150, SE = 0.0048, *p* = 0.0030). This suggests that left UF FA scores partially mediated the relationship between physical neglect and UHR ERT scores, with the combined left UF and physical neglect model accounting for 25% of the variance in UHR ERT scores over the 18% directly observed within the regression.

**Figure 3:**
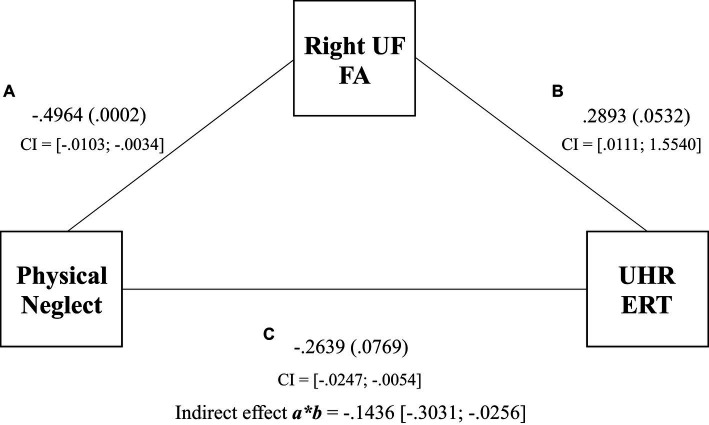
Mediation of physical neglect, right uncinate fasciculus, and the unbiased hit rate total regression coefficients, *p* values, and confidence intervals for associations between physical neglect, FA of the right UF, and UHR total scores. Indirect effect and confidence intervals are represented by a*b.

The results for Model 2 demonstrated a significant indirect effect of physical neglect on UHR ERT via FA in the right UF (β_indirect_ = −0.1436, SE = 0.0708, 95% CI [−0.3031 to −0.0256]) (see Figure 3). Physical neglect was significantly associated with FA in the right UF (β_direct_ = −0.4964, SE = 0.0017, *p* = 0.0002), but the right UF was not significantly directly associated with UHR ERT (β_direct_ = 0.2893, SE = 0.3892, *p* = 0.0532). The total effect of physical neglect on UHR ERT was significant (β = −0.0150, SE = 0.0048, *p* = 0.0030). This suggests that the right UF FA scores fully mediated the relationship between physical neglect and UHR ERT scores. Model 2 accounted for 23% of the variance in the UHR ERT scores.

## Discussion

### Summary of the main findings

The purpose of the present study was to investigate the relationship between the microstructural organisation of the UF and ER outcomes in patients with SCZ. Additionally, we aimed to investigate the sensitivity and specificity of the UHR to control for potential response biases (i.e., guessing), compared to a standard HR, during the ERT (Wagner, 1993). Our study has three main findings. First, moderate negative correlations were observed between physical neglect and the FA of both the left and right UF tracts, independently. Second, moderate positive correlations between FA of both the left and right UF tracts and the UHR ERT were observed, in line with the literature. FA of both the left and right UF significantly predicted ER UHR total scores, where 18% of the UHR variance was accounted for by FA of the left and right UF. Finally, in line with the literature, our mediation analyses demonstrated that physical neglect in childhood was directly associated with UHR performance. There was a significant bilateral partial mediation effect for the UF on the relationship between physical neglect and UHR, accounting for 23–25% of the variance. There was no significant association found for the CST.

### Implications of main findings

Primarily, we find the UHR to be a more significant predictor of performance over the traditional HR on the ERT, and future studies and clinical trials should be considered over the traditional ‘Total Hits’. Further to this, our analyses between the FA of the UF and physical neglect show significant relationships between adverse childhood experiences, WM microstructural organisation, and ER outcomes for people with SCZ. These results further build on the findings by Rokita et al. (2018, 2021), and support previous studies that demonstrated links between FA of the UF and socio-emotional functioning. This would further support links between FA of the UF and ER outside of SCZ (Li et al., 2019; Coad et al., 2020). In the context of SCZ and psychosis-spectrum disorders, these findings are particularly relevant when considering findings from Allott et al. (2014) who demonstrated that poorer ER performance predicted transition to psychosis amongst individuals at ultra-high risk of psychosis. In the present study, bilateral UF FA was predictive of ER performance, and these findings may be important when considering combined predictors for ultra-high-risk individuals and could be an avenue for future research.

Our second series of findings support the previous results from Coad et al. (2020), who report that structural connectivity of the UF tract may be related to the ability to recognise emotion in facial expressions. Coad et al. (2020) reported that FA of the right UF was most related to emotion discrimination ability, and whilst this was not assessed in our study, it is an avenue for future research. This study shows that the UHR score may be more sensitive to capturing ERT subscale recognition. Although the traditional HR score positively correlated with 5/12 subscale emotion items, the UHR score positively correlated with 7/12. Additionally, anger and surprise both demonstrated stronger effect sizes and correlations bilaterally.

Finally, this study shows that childhood physical neglect is significantly associated with bilateral UF microstructural organisation. Furthermore, our mediation analysis shows that childhood physical neglect is not only directly and significantly associated with social cognition, but bilaterally, the UF also partially mediates the relationship. This outcome further supports the neurodevelopmental nature of SCZ in the context of the diathesis-stress model.

### Strengths and limitations

The present study contributes to the literature by providing further evidence of the role of the UF in ER ability. Furthermore, we provide a novel finding that the FA of the UF mediates the relationship between physical neglect and ER. A strength of this study is the use of the UHR score, which provided greater sensitivity to subscale emotion items on the CANTAB ERT (Wagner, 1993; Barnett et al., 2010), accounting for response biases. This study also employed manual tract segmentation, which is considered the gold standard, albeit more resource-intensive, and the sample size of our study was similar to other studies investigating ER and UF FA (Coad et al., 2020).

The use of fibre tractography methods to investigate WM microstructural organisation has several limitations. First, the tensor model can accurately assess fibre tract orientation in regions where fibre populations are isolated and not crossing over (Behrens et al., 2007; Calamante, 2019). However, in regions or voxels where multiple fibre populations intersect, the model fails to accurately assess the multi-directionality of intersecting fibre populations (Calamante, 2019). Additionally, many fibre tracts separate as they arrive at the cortex; therefore, the precision of fibre tractography methods may deteriorate at the point where WM tracts extend out into the cortex (Lazar, 2010). These limitations also extend to the use of FA as a quantitative measure of diffusion anisotropy intended to reflect fibre density (Mukherjee et al., 2008; Figley et al., 2022). For example, regions where various fibre populations intersect make it difficult to interpret and achieve precise measurements that reflect the true fibre density of the tract being extracted (Mukherjee et al., 2008; Figley et al., 2022). A potential limitation of this study is that the efficacy of the CTQ as a measure of childhood trauma may be impacted by the self-report and retrospective nature of the measure (Bernstein et al., 1994). This may facilitate response biases (MacDonald et al., 2016; Gayer-Anderson et al., 2020). Finally, the cross-sectional nature of the study did not allow longitudinal data analysis or for causal claims to be demonstrated or explored.

### Future directions

Whilst previous studies have found that reductions in FA of the UF are specific to SCZ amongst other psychiatric disorders (Koshiyama et al., 2020), other studies have found that UF abnormalities can be directly linked to socio-emotional impairments in neurodevelopmental disorders such as ASD (Li et al., 2019). This may be due to the neurodevelopmental nature of SCZ compared to other psychiatric syndromes, i.e., anxiety, and future research could aim to compare FA across both SCZ and ASD, alongside social cognitive outcomes. Our study found significant differences in the FA of the anterior region of the UF bilaterally, and future research could also investigate this further in relation to clinical and cognitive outcomes. Finally, future studies may consider the use of a UHR total score as a guessing recognition paradigm to account for response biases during ERTs. Our findings demonstrated that the UHR provided greater sensitivity to subscale emotion items, specifically anger and surprise, which demonstrated the strongest correlations to each tract.

## Conclusion

Our findings demonstrate for the first time that FA of the UF partially mediates the relationship between physical neglect and ER, with the model explaining 23–25% of the variance in UHR ERT scores. We observed structural differences between the front of the left UF and the front of the right UF, but not whole tract differences, which may have further implications for frontal syndromes, i.e., dysexecutive function. We found that the FA of the UF predicted UHR ERT performance and accounted for 18% of the variance in UHR ERT scores. Finally, we demonstrated that the use of a UHR is more sensitive to specific subscale items on an ERT task when compared to a standard HR score and should be considered in future studies.

## Data availability statement

The datasets presented in this article are not readily available because data are held in line with the study’s ethical approval. Requests to access the datasets should be directed to the corresponding author, as data are not publically available.

## Ethics statement

The studies involving humans were approved by University Hospital Galway; Tallaght University Hospital. The studies were conducted in accordance with the local legislation and institutional requirements. The participants provided their written informed consent to participate in this study.

## Author contributions

MS: Conceptualization, Formal Analysis, Writing – original draft, Writing – review & editing. SN: Writing – review & editing. EC: Formal Analysis, Writing – review & editing. CE: Writing – review & editing. BH: Writing – review & editing. CM: Writing – review & editing. GD: Funding acquisition, Writing – original draft, Writing – review & editing. TB: Conceptualization, Formal Analysis, Writing – original draft, Writing – review & editing.
